# Chemistry, Inorganic and Organic, with Experiments

**Published:** 1883-12

**Authors:** 


**Chemistry, Inorganic and Organic, with Experiments.** By CHARLES LOUDON BLOXAM, Professor of Chemistry in King's College, London; in the Department of Artillery Studies, Woolwich, etc. From the fifth and revised English edition. Philadelphia: H. C. Lea's Son & Co. 1883.

Bloxam's Chemistry is so well known, both to professional chemists and to medical men, that it is unnecessary to say anything in commendation of the work. The publishers have presented the fifth edition in a way to merit the highest praise. There are nearly three hundred illustrations; and in print, paper and binding, no more could be desired. We find that the accomplished author has made some few alterations in the theoretical portion of the work. A table of atomic weights, arranged so as to indicate the quantivolence of the elementary bodies has been added, and the book is now fully abreast and in harmony with the latest established views. For its size it forms a remarkably complete compendium of chemistry and can hardly be surpassed in the simplicity and clearness of its explanation.

